# Trends in postoperative radiotherapy delay and the effect on survival in breast cancer patients treated with conservation surgery

**DOI:** 10.1038/sj.bjc.6601693

**Published:** 2004-03-02

**Authors:** J Stefoski Mikeljevic, R Haward, C Johnston, A Crellin, D Dodwell, A Jones, P Pisani, D Forman

**Affiliations:** 1 Cancer Medicine Research Unit, Cancer Research UK, St James's Hospital, Leeds LS9 7TF, UK; 2 Northern and Yorkshire Cancer Registry and Information Services, Leeds LS16 6QB, UK; 3 Department of Epidemiology and Health Services Research, University of Leeds, Leeds LS2 9JT, UK; 4 Yorkshire Centre for Clinical Oncology, Cookridge Hospital, Leeds LS16 6QB, UK; 5 School of Environmental Sciences, University of East Anglia, Norwich NR4 7TJ, UK; 6 International Agency for Research on Cancer, cours Albert Thomas, 69372 Lyon, France

**Keywords:** breast neoplasms, radiotherapy, adjuvant treatment, treatment delay, survival

## Abstract

The adequate timing of adjuvant radiotherapy (RT) in breast cancer has become a subject of increasing interest in recent years. A population-based study was undertaken to determine the influence of demographic and clinical factors on the postoperative RT delay in patients treated with breast-conserving surgery (BCS) and to assess the impact of delay on survival. In total, 7800 breast cancer patients treated with BCS and adjuvant RT between 1986 and 1998 in Yorkshire were included in the study. The median interval between surgery and the start of RT (S–RT interval) was 8 weeks (7 weeks for chemotherapy negative and 11 for chemotherapy positive patients). This interval increased substantially over time from 5 weeks during 1986–1988, irrespective of patients' chemotherapy status, to 10 and 17 weeks among chemotherapy negative and chemotherapy positive patients, respectively, in 1997–1998. The S–RT interval was also significantly influenced by travel time to RT centre, year and at which RT centre patient had the treatment (*P*<0.001). Overall, 5-year survival was 82%. Patients with S–RT intervals longer than 9 weeks had a trend towards an increased relative risk of death. This reached a statistical significance at 20–26 weeks (RR 1.49, 95% CI (1.16–1.92)). The findings of our study suggest that delaying the initiation of RT for 20–26 weeks after surgery is associated with decreased survival in patients treated with conservation surgery.

Breast irradiation is now accepted as standard adjuvant therapy following breast-conserving surgery (BCS) (NICE, 2002). Evidence from randomised clinical trials suggests that the use of radiotherapy (RT) is associated with a significantly lower rate of local recurrence in patients treated with BCS (EBCTCG, 2000). Achieving local tumour control is important as relapse often leads to removal of the breast and can result in uncontrolled local disease (Rutqvist, 1996). A causal relationship between local recurrence and distant metastasis has been suggested (Cowen *et al*, 2000), but the meta-analyses and individual clinical trials have so far yielded conflicting results about the survival advantage due to adjuvant RT (Van de Steene *et al*, 2000).

Although there is a common understanding that the effectiveness of adjuvant therapy diminishes with increasing number of clonogenic cancer cells and that such treatment should begin as soon after surgery as is practical (Kurtz, 2002), the maximal safe time interval between breast cancer surgery and the start of RT has not yet been established. Long delays in beginning postoperative RT have been linked to increased risk of local recurrence (Huang *et al*, 2003) but very few studies have looked at the effect on survival. Despite the scarce evidence, concerns about increasing delays in start of RT experienced by breast cancer patients prompted recommendations about the acceptable delays (JCCO, 1993; Clinical Guidelines, 1998; Rutgers, 2001).

We undertook this study in order to investigate the extent of delay in the start of radiation therapy after BCS between 1986 and 1998 in Yorkshire. Our aim was to establish personal, tumour and treatment factors that influenced the postoperative RT delay in breast cancer patients, and examine any relationship between delay and survival.

## MATERIALS AND METHODS

### Study population

All female breast cancer patients diagnosed in the former Yorkshire Regional Health Authority area between 1 January 1986 and 31 December 1998 were identified from the Northern and Yorkshire Cancer Registry and Information Service (NYCRIS) database (*n*=27 941). Patients were excluded from the study if they did not have surgery, had a mastectomy, did not receive RT or had preoperative RT. Cases with known metastatic disease at the time of diagnosis were also excluded from the study.

A case note review of a small sample of patients that had RT later than 6 months after the surgery (*n*=16) indicated that a proportion of them received RT for local recurrence or for palliative purposes. As the RT intent was not available in cancer registry data set for the study period, it was not possible to determine patients who had RT for primary tumour without reviewing case notes of all patients with RT delays longer than 6 months. These cases were, therefore, excluded from the analyses. A review of a sample of 36 case notes of patients who received RT up to 6 months postoperatively showed that the RT was a part of the treatment for the primary tumour in all patients. The total number of cases eligible for the study after all exclusions was 7800.

Patient, tumour and treatment details relevant to the study were abstracted from the NYCRIS database. The pathological (TNM) stage of disease was known for 3781 (48.5%) cases. Based on the information available, an additional 2647 (33.9%) cases were staged: stage I/II (*n*=2126) if cancers were less than 2 cm in size and information about the nodal status was not available, and II/III (*n*=521) if size was >5 cm and lymph nodes were either positive or unknown. For 1372 cases (18%) information available on the NYCRIS database was not sufficient to allow for stage classification; these were included in the analyses as a group of ‘stage unknown’. The Carstairs deprivation index (Carstairs and Morris, 1989) was used as a proxy for socioeconomic status and was determined by the postcode of patients' residence. Estimated car travel time to RT centre was calculated using a Geographical Information System (GIS). These estimates were based on the location of patients and RT centres postcodes, a digital version of the road network, and information on average vehicle speeds over different road types.

Patients included in the study received radiation treatment in one of four RT centres: Leeds (*n*=5290), Hull (*n*=1697), Lincoln (*n*=511) and Middlesborough (*n*=302). Although RT centres in Lincoln and Middlesborough were not within the former Yorkshire Region area, because a considerable number of Yorkshire patients received their RT treatment in these two centres, they were included in all analyses (descriptive and survival) apart from the multivariate analyses of factors associated with the RT delay as they only represented a portion of their case load. These patients were Yorkshire residents and, therefore, may not be representative of the overall population of patients seen at these hospitals. Furthermore, while some of these patients were residing near the border of the region others may have had different reasons why they were referred or chose to go for RT outside Yorkshire and, therefore, results of analyses of the factors associated with the RT delay may be have been biased by including this group of patients.

All patients were divided into six groups according to interval between the date of their surgery (date of first surgery in cases when patients had more than one operation) and the date of start of radiation treatment (1–4 weeks, 5–6, 7–8, 9–12, 13–19 and 20–26 weeks). Separate descriptive analyses were undertaken for patients who received adjuvant CT (CT+ group) and for cases where adjuvant CT was not administered (CT− group).

### Data analysis

#### Factors associated with the RT delay

The influence of the following variables was investigated in relation to the interval between surgery and the start of RT (S–RT interval): age, tumour stage, year of diagnosis, CT, hormone therapy, NHS hospital Trust, RT centre, travel time to RT centre, Carstairs index and whether patient had a second surgical procedure.

After univariate analyses, multivariate linear regression analyses were performed by fitting a model with all variables and all two-factor interactions. Terms were then removed from the model by backward elimination with a threshold for inclusion of a *P*-value of 0.01. The nonsignificant main effect terms were kept in the model if they had a statistically significant interaction with another variable.

#### Survival analysis

The overall 5-year survival for each S–RT category was estimated using the Kaplan–Meier method (Kaplan and Meier, 1958). The survival period for each patient was calculated as the time difference between the date of diagnosis and the death date or censoring date (1 December 2001). Multivariate analysis was performed using the proportional hazards regression model (Cox, 1972). Estimates of the relative risk of death were initially considered for each factor (age, stage, grade, deprivation index, time period, travel time and whether chemotherapy and/or hormone therapy was received) in isolation, and then in a multivariate model using a stepwise approach where each factor was introduced. In the end, only factors that had an impact on the relative risk of dying were left in the model and the relative risks were adjusted for the variation in all those factors.

## RESULTS

The total number of breast cancer patients included in this study was 7800. One-third of the study population (32.4%) were younger than 50 years at the time of diagnosis while 13% were older than 70 years (Table 1

**Table 1:** Female breast cancer patients resident in former Yorkshire Regional Health Authority area, treated with breast-conserving surgery and adjuvant radiotherapy, diagnosed 1986–1998 by age, stage and time period of diagnosis, treatment with chemotherapy and hormone therapy and travel time from home to radiotherapy treatment centre

	**No. of patients**	**% of patients**
*Age* (years)		
<40	755	9.7
40–49	1768	22.7
50–59	2353	30.2
60–69	1931	24.8
70+	993	12.7
		
*Stage*		
I	699	9.0
I/II	2126	27.3
II	3009	38.6
II/III	521	6.7
III	73	0.9
N/K	1372	17.6
		
*Chemotherapy*		
Positive	1857	23.8
Negative	5943	76.2
		
*Hormone therapy*		
Positive	6384	81.8
Negative	1416	18.2
		
*Travel time*		
1–14 min	2224	28.5
15–29 min	3368	43.2
30+ min	2129	27.3
N/K	79	1.0
		
*Time period at diagnosis*		
1986–1988	1308	16.8
1989–1990	903	11.6
1991–1992	1204	15.4
1993–1994	1387	17.8
1995–1996	1404	18.0
1997–1998	1594	20.4

). The median age at diagnosis was 55 years. A large proportion of patients (74.9%) had either stage I or stage II disease (I, I/II or II). A quarter of all cases received CT (*n*=1857, 23.8%) and 82% (*n*=6384) received hormone therapy (Tamoxifen). More than 70% of patients lived within 30 min estimated travel time from the RT centre where they received their treatment (mean travel time was 23 min).

Between 1986 and 1998, the median interval between surgery and the start of RT was 7.6 weeks (53 days, range 8–182 days); 7 weeks (49 days) in the CT− group and 11 weeks (77 days) in the CT+ group (Table 2

**Table 2:** Study patients by time interval between surgery and radiotherapy (S–RT interval) and whether or not chemotherapy (CT) received

**S–RT interval (weeks)**	**CT+ no. of patients (%)**	**CT− no. of patients (%)**	**All no. of patients (%)**
1–4	199 (10.7)	814 (13.7)	1013 (13.0)
5–6	268 (14.4)	1438 (24.2)	1706 (21.9)
7–8	210 (11.3)	1369 (23.0)	1579 (20.2)
9–12	331 (17.8)	1681 (28.3)	2012 (25.8)
13–19	501 (27.0)	579 (9.7)	1080 (13.8)
20–26	348 (18.7)	62 (1.0)	410 (5.3)
All	1857	5943	7800
Median S–RT (days)	77	49	53

). In the first 4 weeks after surgery, 1013 or 13.0% (*n*=814 or 13.7% in CT− group) of patients started their RT treatment.

The S–RT interval increased steadily over the study period. In the earliest time period (1986–1988), the average S–RT interval was about 5 weeks irrespective of patients' CT status (Table 3

**Table 3:** Mean time interval between surgery and radiotherapy by time period and whether or not chemotherapy (CT) received

	**CT+**		**CT−**		**All RT patients**	
**Time period**	**Mean no. of days (95% CI)**	**No. of patients**	**Mean no. of days (95% CI)**	**No. of patients**	**Mean no. of days (95% CI)**	**No. of patients**
1986–88	31.6 (29.8–33.5)	204	36.5 (35.4–37.6)	1104	35.7 (34.8–36.7)	1308
1989–90	42.9 (38.9–46.9)	141	43.2 (41.5–44.9)	762	43.2 (41.6–44.7)	903
1991–92	60.7 (56.1–65.3)	251	50.9 (49.3–52.4)	953	52.9 (51.4–54.4)	1204
1993–94	87.3 (83.2–91.4)	400	57.8 (56.5–59.2)	987	66.3 (64.7–68.0)	1387
1995–96	97.7 (93.9–101.5)	431	61.1 (59.7–62.5)	973	72.3 (70.6–74.1)	1404
1997–98	120.2 (116.8–123.6)	430	70.7 (69.3–72.1)	1164	84.0 (82.3–85.8)	1594

). By 1997–1998, this has increased to 10 weeks among CT− patients (71 days) and 17 weeks in CT+ patients (120 days). The proportion of CT− patients who started their RT during the first 4 weeks was 40% in 1986–1988 in contrast to only 1% in 1997–1998. While only three patients waited 20–26 weeks for the start of their RT between 1986 and 1988, 199 cases (12.5%) waited the same amount of time during 1997–1998.

**Figure 1:**
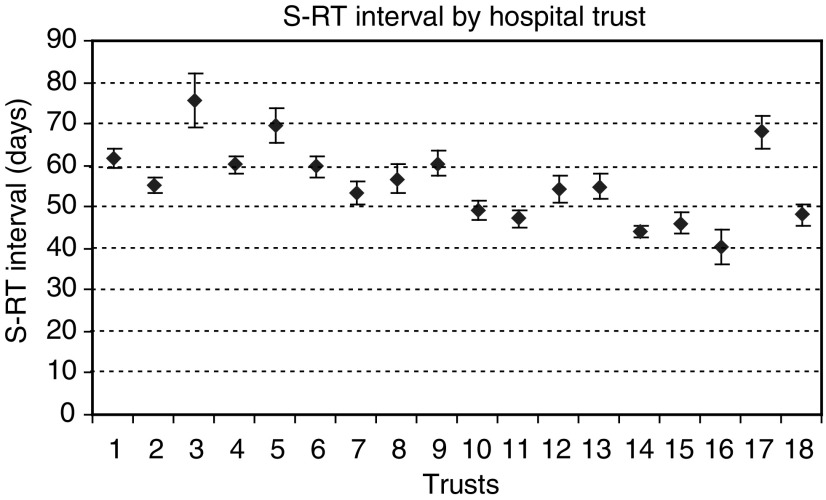
Mean time interval between surgery and radiotherapy by Hospital Trust (chemotherapy negative patients only). 1–10 referred mainly to RT centre A; 11 – private patients; 12–13 referred to either RT centre A or RT centre B; 14–18 referred mainly to RT centre B.

**Figure 2:**
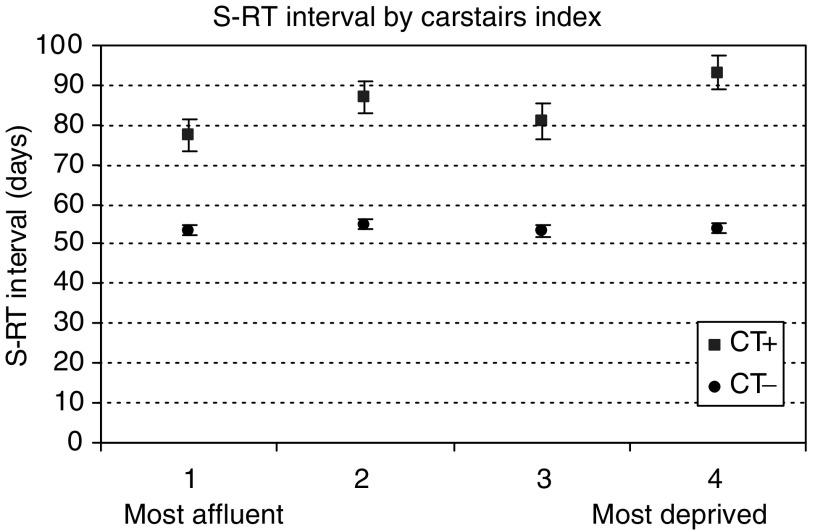
Mean time interval between surgery and radiotherapy by deprivation category and chemotherapy (CT) status.

**Figure 3:**
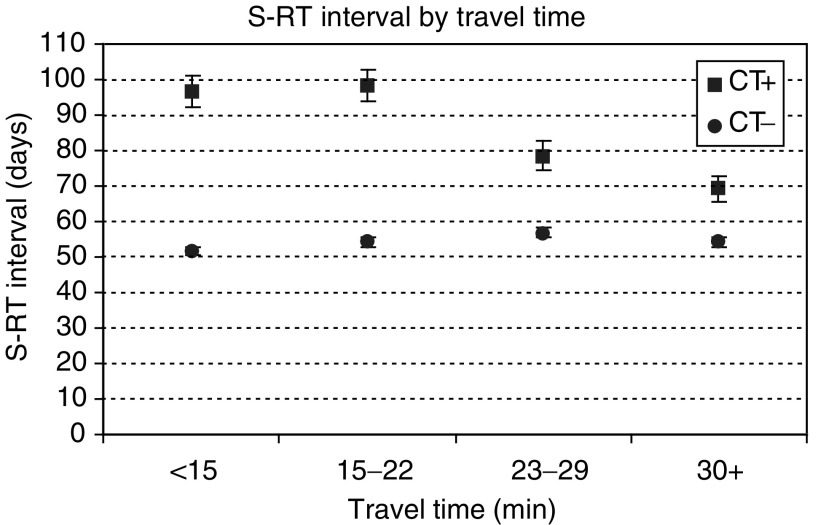
Mean time interval between surgery and radiotherapy by travel time from home to radiotherapy treatment centre and chemotherapy (CT) status.

There was a wide variation between NHS Hospital Trusts in how soon patients started radiation treatment (Figure 1

). For example, CT− patients from three hospitals that referred all their patients to the same RT centre for treatment, had significantly different mean S–RT intervals of 55, 62 and 76 days. Whether patients lived in deprived or affluent areas made little difference to their S–RT intervals if they did not receive adjuvant CT (Figure 2

). However, CT+ patients who lived in more affluent areas had significantly shorter intervals than those who lived in the most deprived areas, 77 days (95% CI 73.36–81.34) and 93 days (95% CI 88.83–97.57) respectively. Travel time to RT centre also made little difference to S–RT intervals in the CT− group but had a significant impact among patients who had CT (Figure 3

). CT+ patients who had to travel longer distances for RT, started their RT sooner than those who lived nearer to the RT centre. Patients who lived within 15 min from their RT centre had on average 4 weeks longer S–RT intervals than those who had to travel more than 30 min (97 and 69 days, respectively).

The results of multivariate analyses of patients' and treatment factors influence on the S–RT interval are presented in Table 4

**Table 4:** Multivariate analysis of demographic, pathologic and treatment factors associated with the time interval between surgery and radiotherapy (S–RT interval)

**Factor**	**Adjusted S–RT interval (days)^a^**	**95% Confidence interval**
*Chemotherapy*		
No	45.6	44.2–46.9
Yes	57.7	55.2–60.3
		
*Stage*		
I	52.5	50.5–54.7
I/II	48.8	50.8–53.2
II	52.0	42.9–58.3
II/III	54.5	52.0–57.0
III	50.0	47.2–50.3
		
*Time period at diagnosis*		
1986–1988	31.2	29.8–32.7
1989–1990	37.8	35.9–39.8
1991–1992	45.5	43.5–47.6
1993–1994	61.2	58.7–63.8
1995–1996	66.4	63.8–69.2
1997–1998	83.3	80.0–86.8
		
*Travel time* (min)		
<15	53.2	51.2–55.2
15–22	53.0	50.8–55.2
23–29	51.5	49.3–53.7
30+	47.7	46.0–49.5
		
*RT centre*		
A	55.3	54.2–56.5
B	47.6	44.8–50.5

a Adjusted for CT, stage, time period, RT centre, Carstairs category and travel time.

. The year of diagnosis, whether patient received CT, tumour stage, RT centre where treatment was received and travel time to RT centre, all had a statistically significant influence on the S–RT interval (*P*<0.001). Significantly shorter S–RT intervals were seen for patients who did not receive CT, those diagnosed in earlier time periods and those living further from the RT centre. Age and Carstairs index were statistically significant in univariate analyses but not in multivariate analysis after the adjustments were made for variation of all other factors. The following interactions had a significant effect on the S–RT interval: year of diagnosis and whether patient received CT, CT and travel time, CT and RT centre and CT and travel time. The statistically significant interaction between time period and travel time showed that in later years there was a trend towards shorter intervals for patients that had longer journeys to RT centre.

The overall 5-year survival was 82.1%. Five year survival by S–RT interval category is presented in Table 5

**Table 5:** Five-year survival and relative risk of death (95% confidence interval)

**S-RT interval (weeks)**	**5-year survival**	**Unadjusted RR**	**Adjusted^a^ RR**
1–4	80.1% (78.5–83.4)	1.00	1.00
5–6	80.1% (78.1–82.0)	0.95 (0.84–1.09)	1.04 (0.91–1.19)
7–8	84.4% (82.6–86.3)	0.81 (0.70–0.93)	0.99 (0.85–1.15)
9–12	84.3% (82.6–86.0)	0.80 (0.70–0.92)	1.04 (0.89–1.22)
13–19	81.0% (78.6–83.6)	0.92 (0.78–1.09)	1.16 (0.96–1.40)
20–26	75.7% (71.1–80.3)	1.25 (1.00–1.55)	1.49 (1.16–1.92)

a Adjusted for age, stage, grade, deprivation index, time period and whether chemotherapy was received (all continuous variables were categorised before analyses; categories were same as presented in Table 1).

Tested also for travel time and whether hormone therapy was received, but were dropped from the final analysis as they did not have significant effect on relative risks of death.

. Women who started RT within first 6 weeks from surgery had a 5-year survival of 80% while it was 76% for patients with a RT delay of 20–26 weeks. The multivariate analyses showed that survival was adversely affected by longer delays (Table 5). After adjustments for the variation in patients' age, stage, grade, deprivation index, time period and whether CT was received, there was a trend towards an increased risk of death in S–RT interval categories longer than 9 weeks. A statistically significant increase in the relative risk of death occurred in patients that had their radiation treatment delayed by 20–26 weeks after operation (RR 1.49, 95% CI 1.16–1.92).

## DISCUSSION

This retrospective analysis of a large population-based cohort of breast cancer patients in Yorkshire who received BCS and RT suggests that the timing of adjuvant RT in breast cancer may be important. Patients who commenced RT treatment between 20 and 26 weeks after surgery had an increased relative risk of death (RR 1.49, 95% CI (1.16–1.92)). These patients had a similar stage distribution but were generally younger than the rest of study population and had a considerably higher CT rate (84.9%). Although only 5% of all patients included in the study started their radiation treatment 20–26 weeks postoperatively, the proportion increased steadily from less than 1% during 1986–1988 to 13% of all patients diagnosed in 1997 and 1998. The trend of increasing delay and the association between RT delay and poorer survival was observed irrespective of the use of adjuvant chemotherapy and persisted after adjustment for stage and age.

Although there is a considerable interest in whether the increasing delays experienced by breast cancer patients before receiving adjuvant RT have adverse effects on outcomes, only a limited number of studies have examined this issue. These have mainly focused on the association with local recurrence, and there are very few published studies of survival. In a small study of 46 breast cancer patients in the US treated with BCS, poorer survival was associated with delays longer than 7 weeks in CT− patients while no significant effects on survival were found in CT+ patients when their radiation treatment was initiated more than 24 weeks postoperatively (Ampil *et al*, 1999). In another US study, breast cancer patients who received RT within 6 months after surgery were compared with patients who had RT postponed for longer than 6 months, and a significantly poorer survival in the latter group was found (Buchholz *et al*, 1993). Both groups of patients had BCS and CT. In contrast to these two studies, in the IBSCG trial, no significant differences were found in the disease-free survival between a 4- or 7-month RT delay period in premenopausal node-positive CT+ women or between a 2- or 4-month period of delay in postmenopausal patients (Wallgren *et al*, 1996). Similarly, no difference in survival was found in CT− patients who had RT delayed for more than 16 weeks after BCS in a Canadian study (Vujovic *et al*, 1998). Huang *et al* reviewed available published studies in this area of studies and their analysis suggested a negative impact of RT delay on local control probability (Huang *et al*, 2003). Guidelines (JCCO, 1993; Clinical Guidelines, 1998; Rutgers, 2001) concerning the timing of adjuvant radiation therapy in breast cancer patients treated with BCS are based on expert advice rather than research evidence.

The effect of treatment delays on outcomes cannot easily be investigated in randomised controlled trials. Observational studies based on high quality routinely recorded data are available for exploring the relationship between therapeutic delay and survival. In this retrospective population-based study, we included breast cancer patients diagnosed within a population of over 3.6 million who had BCS and adjuvant RT, and estimated the relative risk of dying in relation to S–RT interval after adjusting for available patient and clinical factors. The large sample size enabled precision in the estimation of the association between RT delay and survival. While there are limitations of cancer registry data, including incompleteness of tumour stage information and a lack of comorbidity and recurrence information, it is unlikely that they could have biased this large population sample in relation to RT delay. For the 18% of all patients without known stage of disease to bias the survival estimates and be responsible for the effect of decreased survival with long RT delays found in this study, it would require a high proportion of them to be advanced cases who had their RT systematically delayed. However, only a very small proportion (5%) of patients with long S–RT intervals (20–26 weeks) did not have details about their tumour stage. Although comorbidity information was not available, since patients in 20–26 weeks category were generally younger (median 48 years) in comparison to rest of the study population, it is unlikely that comorbidity could explain their poorer survival. Towards the end of the study period new anthracycline-based CT regimens were coming into routine practice that, in contrast to CMF (the previous standard regimen containing cyclophosphamide, methotrexate and 5-fluorouracil), cannot be given concurrently with RT due to increased toxicity. As the new regimens are usually given before RT, there is a possibility that some CT+ patients with the long S–RT intervals in our study population were given anthracycline-based CT regimes. If they were also initially used selectively in patients with poorer prognosis, this would indicate that patients with long RT delays that received CT had worse prognoses than the rest of the study population. Similarly, as patients in the 20–26 weeks interval category were generally younger than patients in other S–RT categories, this might also be an indication of poorer prognoses (as young age is known to be a prognostic factor associated with worse outcomes). In the multivariate survival analyses, we did, however, fully adjust the hazard ratios for variation in age as well as variation in stage, a factor that was known for 82% of all patients (95% of all patients in 20–26 weeks category). Furthermore, patients with more advanced stage of the disease did not have longer S–RT intervals than those with early stages (Table 4). Although information about the type of CT regimens used in CT+ patients was not available, our understanding is that the gradual change to anthracycline-based CT began around 1995/1996 in Leeds, one of the two cancer centres in the region. Since CMF was still the recommended treatment in the national breast cancer guidance published in 1996 (Cancer Guidance, 1996) it is likely that the anthracycline-based CT was not given to patients in the regional district hospitals until later, possibly even after the end of our study period (1998). It was not logistically feasible to review the case notes of nearly 1900 patients diagnosed in 17 regional hospitals in order to determine details of the CT regimens. The consistent trend of increased risk of death in S–RT interval categories longer than 9 weeks would support a more direct link between RT delay and survival, rather than a factor that has not been adjusted for within the study population.

The effectiveness of postoperative RT in preventing or delaying local recurrence in patients who undergo BCS is well established by randomised controlled trials (Liljegren *et al*, 1999; EBCTCG, 2000; Fisher *et al*, 2002). However, meta-analyses and individual clinical trials have produced conflicting results about the survival advantage due to adjuvant RT (Liljegren *et al*, 1999; EBCTCG, 2000; Van de Steene *et al*, 2000; Fisher *et al*, 2002). In the EBCTCG meta-analysis, only a small (nonsignificant) survival benefit was seen in patients randomised to receive RT and BCS compared to those only treated surgically probably because the moderate improvement in breast cancer mortality was counterbalanced by an adverse effect on mortality from cardiovascular causes (EBCTCG, 2000). It may appear that the association between longer delays (20–26 weeks) in the start of RT and inferior survival found in our study contradicts the EBCTCG results since the negative survival impact of delayed RT is of greater magnitude than the negative impact of no RT within the EBCTCG analysis. The findings of this meta-analysis were however strongly influenced by older trials when now redundant RT techniques were used and the risk–benefit relationship may be substantially more favourable with more modern RT practice. A worsening of survival for a small group of patients whose RT is delayed is not incompatible with the conclusions derived from meta-analyses of randomised trials.

Breast cancer patients in Yorkshire experienced increasing delays in the start of adjuvant RT since mid-1980s and delays in the initiation of breast irradiation reported in this study are considerably longer than the current national recommendations. The trend of steadily increasing waiting times for RT following surgery between 1986 and 1998 found in Yorkshire almost certainly reflects the pattern present in RT departments throughout the UK. A small survey of the postoperative RT waiting times for operable breast cancer in the UK indicated that, in 1998, 39% of women with no elective delay waited for adjuvant irradiation more than the maximum target of 4 weeks (RCR, 1998). The greater use of BCS and an increase in referral for RT in addition to inadequate levels of equipment and staff resulted in a steady increase in waiting times for RT across the country. However, due to the significant differences in staffing, beds and equipment levels in RT units within the UK (Department of Health, 1999), the problem is more acute in some regions than in others (RCR, 1998). A survey of RT services in the UK carried out in 2002 indicated that in spite of 5 year national investments in both machines and staff, the RT provision was not sufficient to keep up with the increasing demand for RT (RCR, 2003). During 1997–2002, an increasing number of machines used for planning and delivery of RT were out-of-date and inadequate for the delivery of modern RT and there was still a shortage of suitably trained oncologists, radiographers and physicists, resulting in further increases in RT waiting times. Increasing RT waiting times have also been reported in other countries with similar health care systems (Mackillop *et al*, 1995).

Overall survival has increased over time despite increasing RT delays. Since patients who had RT delayed for more than 20 weeks after surgery represent a small proportion of all patients (5%, *n*=410), their excess risk of dying was not high enough to affect overall survival. The excess risk associated with delayed RT is relative to those that started RT within first 4 weeks, while the trend in overall survival is based on an absolute measure. Survival improved over time among all S–RT interval groups, but patients in the group with longest S–RT delays (20–26 weeks) still had poorer survival.

Radiotherapy treatment for breast cancer involves visits to a RT centre for several days each week over several consecutive weeks. The increased inconvenience of daily travelling to a RT centre for patients who have long journeys can influence to a certain extent the type of surgery they receive, but there is no evidence that it has an effect on RT uptake (Cosford *et al*, 1997; Nattinger *et al*, 2001). Our data suggest that the travel time or distance from RT centre had an influence on how long patients' RT treatment was delayed, with shorter S–RT intervals among CT+ patients who lived further from the RT centre. As mentioned earlier, during the study period anthracycline-based CT regimens were introduced that, in contrast to previous regimens, could not be given concurrently with RT due to increased toxicity. It is possible that patients who lived nearer to cancer centres were managed by specialist teams, including medical oncologists, who adopted the new type of CT regimens earlier than oncologists in other hospitals some distance from cancer centres.

Wide variation of RT uptake between regional NHS Trusts, not explained by the type of surgery performed, has been previously reported (Sainsbury *et al*, 1995). Our findings suggest that a significant variation exists between referring hospitals in how soon patients begin RT. For example, the median S–RT interval in CT− patients in one Trust was 35 days while it was twice as long (70 days) in another Trust. A delay in referral after surgery was previously reported to be the most common reason for delays in patients with S–RT intervals longer than 12 weeks (Vujovic *et al*, 1998). The period under study was before breast cancer multidisciplinary teams were formed and fully operational in most Trusts. With the Breast Cancer Guidance published in 1996 (NHS Executive, 1996), formation of multidisciplinary teams and establishment of their local guidelines for referral and treatment, there is a reason to hope that more equity of access now exists.

Postoperative RT delays have been steadily increasing since mid-1980s in the UK and other countries. It is therefore important to have some understanding of the relationship between postoperative RT delay and adverse treatment outcomes. There is now good evidence that delayed RT increases the risk of local recurrence (Huang *et al*, 2003). Our findings suggest that adjuvant RT in BCS patients should not be unnecessarily delayed and certainly should not commence later than 20 weeks following surgery. Long delays are undoubtedly distressing for patients, increase the risk of local recurrence and may have an adverse impact on survival.
